# The Contribution of Polystyrene Nanospheres towards the Crystallization of Proteins

**DOI:** 10.1371/journal.pone.0004198

**Published:** 2009-01-15

**Authors:** Johanna M. Kallio, Nina Hakulinen, Juha P. Kallio, Merja H. Niemi, Susanna Kärkkäinen, Juha Rouvinen

**Affiliations:** Department of Chemistry, University of Joensuu, Joensuu, Finland; Monash University, Australia

## Abstract

**Background:**

Protein crystallization is a slow process of trial and error and limits the amount of solved protein structures. Search of a universal heterogeneous nucleant is an effort to facilitate crystallizability of proteins.

**Methodology:**

The effect of polystyrene nanospheres on protein crystallization were tested with three commercial proteins: lysozyme, xylanase, xylose isomerase, and with five research target proteins: hydrophobins HFBI and HFBII, laccase, sarcosine dimethylglycine N-methyltransferase (SDMT), and anti-testosterone Fab fragment 5F2. The use of nanospheres both in screening and as an additive for known crystallization conditions was studied. In screening, the addition of an aqueous solution of nanosphere to the crystallization drop had a significant positive effect on crystallization success in comparison to the control screen. As an additive in hydrophobin crystallization, the nanospheres altered the crystal packing, most likely due to the amphiphilic nature of hydrophobins. In the case of laccase, nanospheres could be used as an alternative for streak-seeding, which insofar had remained the only technique to produce high-diffracting crystals. With methyltransferase SDMT the nanospheres, used also as an additive, produced fewer, larger crystals in less time. Nanospheres, combined with the streak-seeding method, produced single 5F2 Fab crystals in shorter equilibration times.

**Conclusions:**

All in all, the use of nanospheres in protein crystallization proved to be beneficial, both when screening new crystallization conditions to promote nucleation and when used as an additive to produce better quality crystals, faster. The polystyrene nanospheres are easy to use, commercially available and close to being inert, as even with amphiphilic proteins only the crystal packing is altered and the nanospheres do not interfere with the structure and function of the protein.

## Introduction

The growth of protein crystals is a complicated process with many variables and hence it is often regarded as being the rate-limiting step in protein structure determination. The crystallization process starts from the formation of critical nuclei by the clustering of molecules in the supersaturated state. In favorable conditions, the nuclei continue to grow and well-ordered, single crystals suitable for X-ray diffraction analysis are obtained. However, finding the favorable conditions for nucleation and crystal growth of the target protein by adjusting parameters such as the precipitant and its concentration, buffer, pH, temperature, and protein concentration (just to mention a few) is a time-consuming task, which still essentially relies on the method of trial and error, in spite of the commercially available crystallization screens that offer an educated initial guess. An additional variable is brought about by the fact that nucleation generally requires a higher level of supersaturation in comparison to optimal conditions for crystal growth, which further complicates the crystallization process. Therefore, methods have been sought to uncouple the two procedures, in other words, to evoke heterogeneous nucleation.

Seeding (thorough review by Bergfors 2003 [1]) is a method commonly used to initiate crystal growth by introducing nuclei to a crystallization drop at a lower level of supersaturation. This approach is usually applied when crystals of inadequate quality or crystalline precipitate are obtained from initial crystallization trials. The seeding method is taken one step further in micro-seed matrix-screening [2], [3], where crystal seed-stock is obtained from one condition and then used to seed a crystallization screen. However, these techniques build on the existence of some kind of crystalline material, which is not always the case. Thus, the pursuit of a universal heterogeneous nucleant for protein crystallization continues.

Heterogeneous nucleation is sometimes introduced into the crystallization experiment by accident in the form of an eyelash or a hair of the experimenter. Nucleation using natural materials, such as human and horse hair, rat whiskers and dried seaweed, has been systematically studied [4]–[6], and appears to promote nucleation in the metastable zone, when applied to protein standards (such as lysozyme and xylose isomerase), as well as with proteins under study. The presence of the native protein surface (keratin) in the nucleating hair was found to be crucial for the nucleation event and crushing the hair also had a favorable affect.

A deliberate introduction of a foreign object into the crystallization drop has also been explored with minerals [7] so as to generate epitaxial crystal growth, and with gel-class [8], a porous media with pore sizes related to the size of the protein. The experiments by McPherson and Shlichta were one of the first systematic studies of heterogenic nucleation. Yet, this method has not attracted a vast number of crystallographers as users, most likely due to an excess amount of minerals to choose from, since a specific mineral suitable for nucleating any protein has not been identified. In the study of the gel-class as a nucleant, all the test proteins were crystallized in the metastabile zone. The control experiments produced no crystals and drops with nucleants of unsuitable pore size remained clear. The study concluded that a universal nucleant might be a disordered porous medium with an ununiform pore size. In this way, the protein molecules could cluster to a pore of suitable size and promote nucleation.

Luring the protein molecules to cluster and hence form nuclei for crystal growth has also been attempted, by modifying the surface the crystallization drop is in contact with. Polymeric films that contain ionizable groups (such as sulfonated polystyrene) have been tested for this purpose [9] and this approach was found to shorten the crystal growth time and to lower the protein concentration required. Film techniques were also used in the crystal production phase of a new approach for protein structure determination, which is termed protein nanocrystallography [10]–[12]. Here, the Langmuir-Blogett technology is used to create an ordered film, composed of the target protein, on the surface of the cover slide of the crystallization experiment. It was shown, that molecules are actually transferred from the LB film to the crystal and that the presence of the LB film does promote nucleation.

In search of the universal nucleant, we looked for a nucleant that is at hand or convenient to purchase, simple to use, effective, and also chemically inert. The efficiency or nucleating power is naturally the most important criterion. However, a clever technique might be in danger of being discarded if the process or the tools are too complicated i.e. require large and expensive equipment or the method simply has too many variables involved. Chemically inert substances are attractive, since they are unlikely to affect the state of the research target. Bearing in mind the above criteria, nanospheres made of polystyrene appeared to be appealing candidates for nucleation of protein crystals. Previously, nanoparticles of different composition have been found to enhance nucleation, and, consequently, the fibril formation of β_2_-microglobulin, a human protein, the self-assembly of which is involved in dialysis-related amyloidosis [13].

We chose lysozyme, xylose isomerase and xylanase as our target proteins and Crystal Screen HT as the test case screen because they are widely used for testing and commercially available. Hydrophobins, laccase, SDMT and 5F2 are real life research targets and included in this study to investigate whether or not nanospheres are also of use in a non-standard experiments. In this study we found that nanospheres in combination with a commercial crystallization screen increase the crystallization success when screening crystallization conditions and that nanospheres can also be used as an additive in the fine-tuning of crystallization conditions, especially for problematic crystals.

## Results

### Screening

The nanosphere screening experiment with HEWL yielded well-shaped, single crystals from nine conditions (out of 96 conditions in Crystal Screen HT) and spherulites or needles from three conditions. It was difficult to distinguish between the precipitating protein and the precipitate due to the nanospheres (the nanosphere solutions appeared milky and nearly always left the droplet unclear), hence the precipitation of the protein could not be evaluated. However, the HEWL control experiment (water added to a crystallization screen instead of nanosphere solution) yielded single crystals from four conditions only and spherulites from one. A granular precipitate, possibly crystalline, was present in several drops. Four crystal-producing conditions were common to both the nanosphere and control experiment, one was unique to the control screen and eight were unique to the nanosphere screen. Drops containing crystals in the nanosphere screen but not in the control screen remained clear, excluding one condition that yielded precipitate in the control screen.

To evaluate the repeatability of the experiment, the screening experiment was repeated for HEWL two months later. The results were similar but not the same. Single crystals were obtained from nine conditions in the nanosphere screen and from three conditions in the control screen. Spherulites grew from three conditions in the nanosphere screen and two conditions in the control screen. Two conditions producing crystals was common to nanosphere and control screens, as well as one condition that produced spherulites. In comparison to the previous screening experiments, the crystal producing conditions varied considerably, both in the nanosphere screen and the control screen i.e. only three of the crystal producing droplets gave exactly the same result as two months before.

For xylose isomerase, an abundance of crystals formed in both the nanosphere and the control screen. 32 conditions produced crystals in the nanosphere screen and 29 conditions in the control screen. Seven conditions were unique to the nanosphere screen and five to the control screen. Spherulites were found in two droplets, both in the nanosphere screen and in the control screen; one of the conditions was common to both screens.

Xylanase produced crystals in eight conditions in the nanosphere screen, three of which were also found to be crystalline in the control screen. One of the droplets in the control screen produced crystals, in addition to those three conditions common to the nanosphere screen.

Table 1 summarizes the results of the screening experiments. Thus, the presence of nanospheres in the screen always produced more crystals in comparison to the control screen. 12.5%, 12.5%, 35.4%, and 8.3% of the droplets contained crystalline material in the nanosphere screens of HEWL1, HEWL2, XI and XYNII, respectively, whereas the control screens contained crystalline material in 5.2%, 5.2%, 32.3%, and 4.2% of the droplets. The results were similar in both HEWL screens and the XYNII screen, where roughly 10% of the droplets produced crystals in the nanosphere screens and about 5% in the control screens. However, in the case of XI, both the nanosphere screen and the control screen had over a 30% success rate in crystallization. No clear tendency was observed in the nanosphere screening experiments for certain types of conditions (*e.g.* solutions containing polyethylene glycol) being favorable for crystal growth in the presence of nanosphere.

**Table 1:** Summary of the effects of polystyrene nanospheres in the screening experiment with lysozyme, xylose isomerase, and xylanase in combination with Crystal Screen HT.

Protein	Control	Nanosphere 50 nm	
		+	−
Lysozyme (HEWL)	5	8	1
Lysozyme (HEWL, parallel)	5	9	2
Xylose isomerase (XI)	31	8	5
Xylanase (XYNII)	4	5	1

Lysozyme experiment was repeated in order to assess the repeatability and therefore appears in the table twice. Numerical value indicates how many conditions produced crystals out of 96 possible conditions in Crystal Screen HT. Control experiment contained equal amount of water instead of nanosphere solution. ‘+’ indicates crystals in the nanosphere screen only, not in the control screen. ‘−’ indicates conditions that produced crystals in the control screen but not in the nanosphere screen.

**Figure 1:**
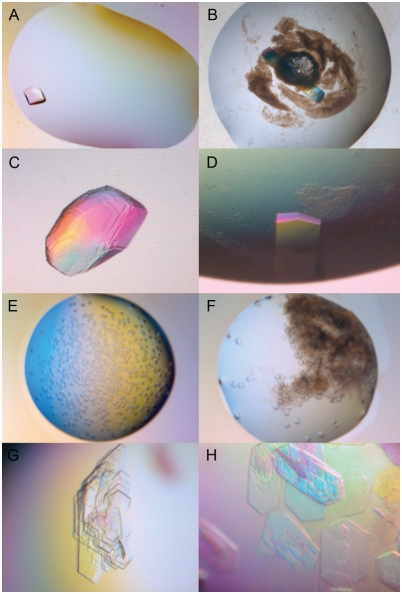
Examples of the effects of the nanospheres on crystallization in the screening experiments of lysozyme (A–D), xylose isomerase (E, F), and xylanase (G, H) in combination with Crystal Screen HT. Crystals from control screens (A, C, D, G) and nanosphere screens (B, D, F, H) are shown. Precipitate due to nanosolution is present in B and F.

Some of the crystals, produced in the same conditions, visually appeared in different shapes (Figure 1 A, B), according to whether they originated from the nanosphere screen or control screen. In most cases, however, the crystals appeared alike in both screens. Representative crystals from the screens were tested with X-ray diffraction and the space group and the cell parameters were always almost identical, even if the crystals appeared to be different by visual inspection. The diffraction power of the crystals was also alike in most cases. However, in, for example, the case of condition C11 of Crystal Screen HT (0.1 M HEPES sodium pH 7.5, 0.8 M sodium phosphate monobasic monohydrate, 0.8 M potassium phosphate monobasic) in the first lysozyme experiment, the crystal from the nanosphere screen diffracted to 1.66 Å whereas the crystal from the control screen appeared to be disordered both visually and when judged by the diffraction pattern and diffracted to 3 Å only (Figure 1 C, D). Sometimes the crystals in the nanosphere screen were fewer in number and larger in size in comparison to those in the control screen (Figure 1 E, F), and vice versa, thus no clear tendency on the effect of the nanosperes in relation to the size or number of the crystals in the screening experiment was found. However, in three cases the crystals in the nanosphere screen were singular while they grew in a bunch in the control screen (Figure 1 G, H).

### Hydrophobin HFBII

**Figure 2:**
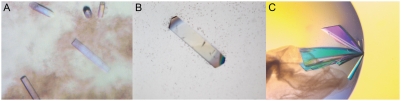
Crystals of HFBII (A), HFBI (B), and SDMT (C) produced with nanospheres.

Hydrophobins were selected as target proteins especially because they are amphiphilic proteins and contain a hydrophobic patch on the protein surface, and hence could presumably interact with the hydrophobic moiety of the polystyrene nanospheres. Using the nanospheres as additives in the known crystallization conditions of HFBII yielded crystalline material with each nanosphere diameter that was tested (*i.e.* 20 nm, 50 nm, 100 nm, and 500 nm). The crystallization experiment, containing a control drop, dilution series and the stock solution for each nanosphere size, was pipetted twice. Even though the concentration of the precipitant was lowered in order to avoid crystallization in the control droplets, crystals were obtained on one third of the control drops. The crystals in the control drops were of spherical or irregular shape, while the crystals in the nanosphere containing drops were shaped like thin needles or were rectangular. The largest, rectangular crystals grew with stock solution of 50 nm nanosphere (Figure 2 A). This experiment was repeated six times with identical results. Data were collected on one of these crystals to a 1.9 Å resolution at the EMBL Hamburg, beamline ×12. The crystals in the control drops were disordered and data could not be collected. However, previous data collection of ordered crystals from the same conditions were of space group I23 with unit cell dimensions a = b = c = 72.2 Å and a resolution of 3.1 Å.

HFBII has also been previously crystallized in a visually similar crystal form, as in the nanosphere experiment. The rectangular crystals of HFBII may be obtained from the same conditions used in the nano experiment, by streak-seeding and addition of manganese chloride. However, these crystals, grown in the presence of manganese belong to space group C2 with unit cell dimensions of a = 78.7 Å, b = 46.3 Å, c = 34.6 and β = 112.2°. The crystal grown in the presence of the nanospheres (and in the absence of Mn-ions) was of orthorhombic space group I222 with unit cell parameters a = 42.1 Å, b = 91.4 Å, and c = 94.8. The crystal grown with nanospheres was partially pseudo-merohedrally twinned (twin fraction α≅0.3), which is possible in the orthorhombic space groups when the b- and c-axes are approximately equal, which is the case here. However, since the twinning was not perfect, it did not affect the data processing by mimicking a higher space group. The twinning was accounted for in the refinement performed with SHELXL with twin operator *h*, *l*, *−k*.

The data was refined to final R-values of R = 19.8 and R_free_ = 25.6. The structure is described in detail in a separate publication (J.M. Kallio *et al.* manuscript in preparation). The packing of the molecules in the crystal was found to be divergent of those previously reported for HFBII [14], [15] and additional electron densities were observed close to the hydrophobic surface areas of the protein in the crystal structure. This density clearly fitted a small molecule with an aromatic ring and was thus concluded to have originated from the nanosphere solution. A styrene monomer (possibly present in residual amounts in the nanosphere solution) could be placed in the density.

### Hydrophobin HFBI

The use of detergent has a significant effect on the diffraction power of hydrophobin HFBI crystals and therefore the nanosphere experiments were conducted both in the presence and in the absence of the OSG-detergent. In the absence of the detergent, small crystals for control drops and for nanosphere drops in which the nanospere solution had been diluted 1∶49 or 1∶19 with water were produced. The crystals did not diffract with the home source and the appearance of the crystals was the same in both the control and the nanosphere drops. In the nanosphere experiment with the detergent present in the presence of the detergent, small crystals in the shape of a parallelogram or a rectangle were obtained with each solution of nanospheres of different sizes and different dilutions. Data were collected for a tiny crystal grown with a 1∶49 dilution of 100 nm nanosphere at the ESRF Grenoble, beamline ID29 but were found to represent exactly the same crystal form as previously reported [16]. The crystallization conditions were further optimized and from a droplet, containing 100 nm nanospheres with a dilution of 1∶9 in water and the protein concentration elevated to 8 mg/ml, a rectangular crystal was grown (Figure 2 B). This experiment was repeated 18 times with alike results. Tested with the home source the crystal diffracted to about 3.5 Å and appeared to be of an orthorhombic crystal form with unit cell dimensions a = 49.7 Å, b = 131.1, and c = 114.4 Å, which differs from the crystal form previously observed for HFBI [16]. However, due to the low resolution and icing of the crystal the data were not collected.

#### Laccase

rMaL usually crystallizes as clusters of small crystals due to excessive nucleation, which also causes non-merohedral twinning, disorder and other crystal defects. Optimization of crystallization parameters and the use of many additives to control the nucleation and crystal growth have been tried. So far, single laccase crystals have only been achieved by streak-seeding. By introducing the seeds of crystals grown from 15% PMME2000 into the new drops equilibrated at lower levels of supersaturation, large single crystals that even diffract to 1.3 Å [17] can be grown.

**Figure 3:**
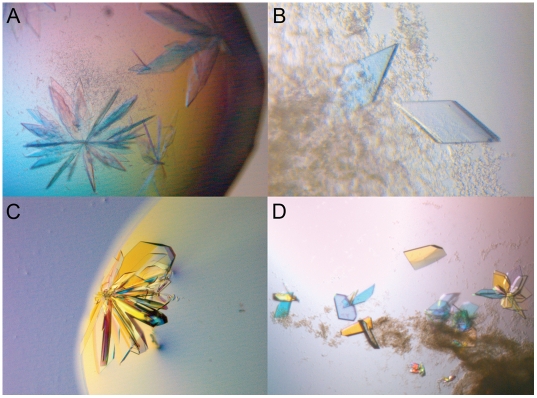
Nanospheres in combination with streak-seeding. Laccase (A, B) was crystallized with nanospheres as an alternative for seeding, whereas seeding was used in combination with nanospheres for 5F2 Fab fragment (C, D). Both laccase and 5F2 grew in bunches (A, C) and single crystals were obtained with the use of nanospheres (B, D).

When 15% PMME2000 was used as a precipitant, all the nanosphere sizes tested produced crystals. In particular with 20 nm nanospheres many small but good quality single crystals instead of clusters, as seen in the control tests (Figure 3 A, B) were obtained. By reducing the precipitant concentrations to 13%, no crystals were detected in the control tests, but large crystals were grown with the 20 and 50 nm nanospheres. These experiments with stock solutions of nanospheres were triplicate. In addition, the effect on dilution of nanospheres was tested. The experiments including the control drop, dilution series and the stock solution of each nanosphere size were pipetted at least twice for rMaL. The tendency in the results was such that the 100 nm and 500 nm nanosphere sizes rarely produced crystals while with 20 nm and 50 nm nanospheres (non-diluted or 1∶1 diluted), crystals were nearly always present. Some of these crystals were large and single, having the same space group, very similar unit cell dimensions and similar kinds of diffraction power to single crystals grown by streak-seeding.

### Sarcosine dimethylglycine N-methyltransferase

SDMT crystals typically grow as rather large bundles, from which single crystals may be separated for X-ray measurements. When a nanosphere solution was added to the crystallization drops, visible crystals could be detected in two days whereas crystals in the control droplets grew within one week. In addition, the quantity of the crystals in a single drop was less and the crystals were larger and visually of better quality than in the control drops. However, mostly the crystals still grew in bundles (Figure 2 C) although some single crystals grew in the presence nanospheres.

Control drops, various dilutions (including 1∶1, 1∶4, 1∶19, and 1∶49) and stock solutions of 20 nm, 50 nm, and 100 nm nanospheres were pipetted in duplicate for SDMT. Crystals grew from all the solutions of 20 nm and 50 nm nanospheres, however, the best results were gained with slightly diluted (1∶1 to 1∶4) solutions. The use of a stock solution of nanospheres led to the formation of a strong precipitate, which complicated the crystal handling. More diluted solutions diminished their effect on the crystallization causing crystals to grow as several small bundles, similar to control drops.

Crystals formed with nanospheres were tested by using synchrotron radiation at EMBL/Hamburg on beamline ×12 and crystals diffracted to a maximum of 1.8 Å. The use of nanospheres did not have any effect on the diffraction power of crystals nor did it alter the crystal packing.

### Anti-testosterone Fab fragment 5F2

5F2 crystals grow in clusters from which the single crystals cannot be separated. Streak-seeding is an effective method for producing single crystals, however, crystals suitable for X-ray analysis are obtained only when the crystallization droplets are allowed to equilibrate for a relatively long time (approximately three weeks) before seeding. In the nanosphere experiment, nanospheres were added prior to seeding to the crystallization droplets, which were seeded one, four, seven or 14 days after the preparation of droplets. Crystals grew with each nanosphere size, however, the best results were gained with 50 nm nanospheres with 1∶1 dilution. This experiment was repeated 30 times and always produced similar crystals. Results were compared to control droplets, which contained an equal volume of pure water instead of nanospheres. Regardless of the equilibration time, single and well-ordered crystals grew along the streak line in all nanosphere droplets. Small, growing crystals were visible as early as two hours after the seeding. In contrast, crystals did not grow in the control droplets with equilibration times of one and four days. Several weeks later some crystal clusters appeared away from the streak line, as a result of spontaneous nucleation. When the control droplets were allowed to equilibrate for seven days, crystal clusters grew on the streak line. Single crystals were obtained from the control experiment only when the equilibration time was 14 days.

Single crystals were only obtained by streak-seeding, otherwise the crystals grew in clusters, also in the nanosphere droplets (Figure 3 C, D). Nanospheres combined with the streak-seeding method yielded a crystal that diffracted to 1.5 Å resolution on beamline ID29 at the ESRF, Grenoble. Data were collected and structure determination is in progress (M.H. Niemi, unpublished results). Data has not been collected on crystals grown without the nanospheres.

## Discussion

### Practical aspects of using nanospheres

The nanosphere solutions appeared like milky emulsions rather than clear aqueous solutions. Nanosphere solutions also needed to be thoroughly shaken before pipetting to insure they are well suspended. However, the solutions were not uncomfortable to pipette nor did they clog the pipette tip. In many occasions, the use of nondiluted stock solutions in crystallization was feasible, in spite of the large amount of precipitate formed in the crystallization droplet by the nanospheres. In some cases, for example SDMT, the use of a diluted nanosphere solution produced better results, as the precipitate due to the stock solution of nanospheres stuck to the crystals. The precipitate from the nanosphere solution was more intense on larger nanospheres (100 nm and 500 nm). However, if too diluted (1∶49, 1∶19), the nanospheres no longer produced the desired effect on the crystallization, as was found for HFBI.

### Size scale

In using the nanospheres as additives the largest, most ordered, singular crystals grew with 20 nm (rMaL and SDMT), 50 nm (HFBII, rMaL, SDMT, and 5F2), and 100 nm (HFBI) nanospheres. The tested proteins were 3–7 nm in diameter [14], [16], [18]. The shape of the crystals produced with the nanospheres ranged from thin plates to rectangular crystals and the crystal dimensions from 5000–1 000 000 nm. As protein crystals regularly contain 27–78% water [19], large solvent channels pass through the crystals. However, these channels are typically not large enough to allow a flow through of nanospheres, at least not nanospheres in the size scale used in this study. However, we cannot rule out the possibility of nanosphere(s) being incorporated into the crystal structure, possibly as nucleant(s). Most importantly, however, the nanospheres did not interfere with the diffraction of the protein nor did they complicate the data collection or structure determination.

### Structural effects

The use of nanospheres altered the crystal packing of the hydrophobin HFBII and caused it to crystallize in another space group. Also hydrophobin HFBI seemed to crystallize in a new space group, even though this was not evaluated due to the low resolution of the data. Detergents as additives also change the space group in which HFBI and HFBII crystallizes [15], [16]. However, no corresponding changes caused by the nanospheres were observed for other proteins investigated in this study. Hence, it may be concluded that the effects of nanospheres on the crystal packing of hydrophobins are due to the peculiar properties of hydrophobins i.e. their amphiphilic nature and tendency to produce various structural assemblages. This has encouraged us to think that the nanospheres could also be used in the crystallization of proteins with hydrophobic surface areas, such as amphiphilic proteins and membrane proteins. The presence of nanoparticles could affect the packing of these molecules, however, the fold and the native structure of the protein would not be altered.

### Nucleation

In each screening experiment, the nanosphere screen always produced more crystals, indicating that nanospheres enhance nucleation. In the case of the xylose isomerase, nucleation seemed to be in abundance in either case and the effect of nanospheres was diminished. However, in most cases, the crystallizing conditions were not common to both the nanosphere screen and the control screen and some unique conditions were also found in the control screen. This indicates that the nanospheres might have a retarding or even an inhibitory effect on crystallization in some cases. This property is a drawback for nucleation but could, nevertheless, be exploited when the excess of nucleation, rather than the lack of it, is a problem and cannot be controlled by any other means. As in the case of laccase, nanospheres could be used as additives in crystallization to control nucleation and to improve crystal quality as an alternative for seeding. Also, the combination of seeding and the use of nanospheres in the crystallization of 5F2 and crystallizing SDMT with no seeding involved produced better quality crystals and sped up crystal growth, as well.

In order to compare the nucleating power of nanopheres to previously studied nucleating agents we compared our results to those of Thakur and co-workers [6]. They have recently studied the effects of fumed silica, CM sephadex, sand, titanium (IV) oxide, glass wool, hydroxyapatite, celluose, horse hair and dried seaweed as nucleants in combination with nine proteins (including lysozyme, xylose isomerase and xylanase) and Crystal Screen HT. They found dried seaweed, horse hair, cellulose and hydroxyapatite to be the most effective nucleants, each producing 0–4 new crystallizing conditions for the studied proteins and causing the loss of 0–2 conditions. Our study is not fully comparable to Thakur and co-workers due to different experimental set-ups. Yet, nanospheres do seem quite effective nucleants as they produced 5–9 new crystallizing conditions and resulted to the loss of 1–5 conditions.

Our study clearly indicates that the use of nanospheres may induce both an enhancement and a retardation effect on nucleation, depending on which is desired. What is less clear, however, is how this occurs. The increase in the nucleation could proceed either a) by the nanosphere itself acting as a heterogeneous nucleant or b) through the clustering of protein molecules closer together (due to the interfering effect of nanospheres, which take up space in the solution) and hence the formation of critical nuclei. These two alternatives cannot be distinguished between by visual inspection (whereas epithaxial growth on mineral surface or nucleation caused by an eyelash in the crystallization drop can be observed under microscope) because nanospheres are truly in nanoscale and individual spheres cannot be discerned on crystal surface. According to the manufacturer, the nanospheres are spherical and smooth, which rules out the possibility that some protein molecules might cluster in pores or grooves on the particle surface.

What remains to be tested are the effects of alternative compositions, shapes and size-homogeneity of the nanoparticles used for crystallization. Nanoparticles made of different materials are commercially available in abundance. Different shapes or material containing various shapes also exist, as well as nanoparticles with a wide size distribution instead of homogenous particle size. As we continued to experiment with nanoparticles of various kinds, the effects of nanoparticles on crystallization and the growth mechanism will be further clarified.

## Materials and Methods

### Protein materials

The proteins used in the crystallization trials were Hen Egg White Lysozyme (HEWL), *Trichoderma reesei* xylanase (XYNII), *Streptomyces rubiginosus* xylose isomerase (XI), *T. reesei* hydrophobins HFBI and HFBII, recombinant *Melanocarpus albomyces* laccase (rMaL), recombinant sarcosine dimethylglycine N-methyltransferase (SDMT) originally from *Halorhodospira halochoris*, and anti-testosterone Fab fragment 5F2 isolated from a naïve human phage display library. HEWL, XYNII and XI are standard laboratory proteins available commercially [20]–[22], with molecular mass of 14.6 kDa, 21 kDa, and 173 kDa for HEWL, XYNII and XI, respectively. Hydrophobins are small, about 7 kDa, amphiphilic proteins produced by filamentous fungi and contain hydrophobic surface areas. Laccase is a multicopper oxidase of 72 kDa in size. SDMT is a 32 kDa enzyme that catalyzes the two-fold methylation of sarcosine to glycine betaine, with S-adenosylmethionine (AdoMet) as the methyl group donor. Fab fragment 5F2 contains the antigen binding part of an antibody and the molecular mass of 5F2 is approximately 50 kDa.

HEWL was purchased from Sigma Aldrich and dissolved in pure water to a concentration of 20 mg/ml for the crystallization trials. Xylose isomerase and xylanase were produced by Macrocrystals Oy and delivered as crystalline suspensions. Xylose isomerase was made soluble by a three-day dialysis with pure water, after which the concentration of the protein solution was determined to be 20 mg/ml by A_280_. Xylanase was centrifuged and washed with water and finally made soluble with a 40% solution of glycerol in pure water. The concentration of protein solution was determined to be 10 mg/ml by A_280_. Hydrophobins were produced and purified, as previously described [23], [24], at the VTT Technical Research Centre of Finland. The lyophilized protein materials were then dissolved into pure water to a concentration of 4 mg/ml and 8 mg/ml of HFBI and HFBII, respectively. rMaL laccase was expressed in *T. reesei* and purified, as previously described [25], at the VTT Technical Research Centre of Finland. A laccase concentration of 8 mg/ml (determined by BioRad) was used. SDMT was produced recombinantly in *Escherichia coli* and purified, as described previously [26], in the Laboratory of Bioprocess Engineering in the Helsinki University of Technology. The concentration used in the crystallization was 13.5 mg/ml. Fab fragment 5F2 was isolated, produced and purified at the VTT Technical Research Centre of Finland (unpublished data). For crystallization, the protein concentration was 8.3 mg/ml.

### Nanospheres

The nanosphere size standards were purchased from the Duke Scientific Corporation, where they are manufactured for use as a calibration standard for electron and atomic force microscopy. The nanospheres are available in multiple diameters, of which the sizes 20, 50, 100 and 500 nm were tested in our experiments. The certified mean diameters of the nanospheres, as reported by the manufacturer are 21 nm±1.5 nm, 50 nm±2.0 nm, 102 nm±3 nm, and 499 nm±5 nm for 20 nm, 50 nm, 100 nm, and 500 nm spheres, respectively. The particles are composed of polystyrene and delivered as aqueous suspensions with a density of 1.05 g/cm^3^.

### Crystallization setups

The method employed for the crystallization was hanging-drop vapor-diffusion at room temperature. Crystallization trays were set up manually using Greiner Bio-One sterile Cellstar 24-well TC-plates. The volume of the reservoir solution was 500 µl and the drop volume ranged from 5 to 10 µl. Unless stated otherwise further in the text, the crystallization drop contained 10% (v/v) of nanosphere solution, 50% (v/v) protein solution and 40% (v/v) of reservoir solution, which was composed of the precipitant(s) and the buffer. In order to make comparison, control drops were also set up for each experiment. This means that instead of the nanosphere solution the droplet contained an equal volume of pure water.

### Nanospheres in the crystallization screening

The use of nanospheres in conjunction with the screening of the crystallization conditions was tested with HEWL, XYNII and XI by using the 50 nm nanosphere solution and the Crystal Screen HT solutions, purchased from Hampton Research. For HEWL, a parallel screening test was also performed in order to assess the repeatability of the experiment. All the screen tests were visually observed for three months in order to ensure that excess time was given for crystallization to occur.

### Nanospheres as additives

The effect of nanospheres as additives for known crystallization conditions were tested with HFBI, HFBII, rMaL, SDMT and 5F2. Also, dilution series of each nanosphere size (1∶49, 1∶19, 1∶4, 1∶1) in water were prepared so as to examine the effects of the concentration. In these experiments, the crystallization drop contained 10% (v/v) of diluted solution of nanospheres.

The known crystallization condition for HFBII is 30% (w/v) polyethylene glycol (MW 2000), 0.2 M lithium sulphate and 0.1 M Tris-HCl at pH 8.5 [27]. A similar solution, with the PEG-concentration lowered to 15%, was used for the nanosphere experiments because this is the metastabile zone of HFBII, as previously determined in streak-seeding experiments.

Hydrophobin HFBI was previously crystallized with 0.1 M zinc sulphate and 0.1 M sodium cacodylate at pH 6.5 [16]. The detergent 1-s-octyl-β-D-thioglucoside (OSG) was used as an additive with its concentration in the crystallization drop being 9 mM. The use of detergent improved the diffraction power of the crystals from 9 Å to 2.1 Å. Nanosphere experiments were carried-out for HFBI with and without the OSG-detergent. When the detergent was not present, the concentration of zinc sulphate was lowered to 0.05 M in order to reach the metastabile zone. With the detergent present, the crystallization drop contained 2 µl of precipitant-buffer solution, 0,5 µl of nanosphere solution, 0,5 µl of OSG and 2,5 µl of protein.

rMaL was crystallized, as previously described, with 13% (w/v) polyethylene glycol monomethyl ether (MW 2000), 0.1 M ammonium sulphate and 0.1 M sodium acetete at pH 4.4 using streak-seeding [18]. In the case of laccase, the crystallization droplets contained 2 µl of crystallization solution, 2 µl of protein solution and 1 µl of the nanosphere solution.

For SDMT the concentration of the nanosphere solution in the droplets was altered from 20% (v/v) to 10% (v/v), maintaining the concentration of reservoir solution added to the droplets as constant (40% (v/v)). The crystallization solutions for SDMT contained 15% (w/v) polyethylene glycol (MW 3350), 0.1 M magnesium, calsium or strontium dichloride and 0.1 M HEPES at pH 7.5 (J.P.Kallio, unpublished results).

For the crystallization of 5F2 Fab fragment, the droplets were prepared by mixing 2 µl of 5F2 protein solution, 0.5 µl of testosterone solution (5 mM in 50% ethanol), 1 µl of nanosphere solution and 2 µl of precipitant solution containing 12% (w/v) polyethylene glycol (MW 3350) and 0.1 M sodium citrate at pH 4.7 (M.H. Niemi, unpublished results).
